# Reward learning and negative emotion during rapid attentional competition

**DOI:** 10.3389/fpsyg.2015.00269

**Published:** 2015-03-12

**Authors:** Takemasa Yokoyama, Srikanth Padmala, Luiz Pessoa

**Affiliations:** ^1^Department of Psychology, University of MarylandCollege Park, MD, USA; ^2^Graduate School of Environmental Studies, Nagoya UniversityNagoya, Japan; ^3^Japan Society for the Promotion of ScienceTokyo, Japan; ^4^Neuroscience and Cognitive Science program, University of MarylandCollege Park, MD, USA

**Keywords:** reward learning, negative emotion, attention, motivational significance, affective significance

## Abstract

Learned stimulus-reward associations influence how attention is allocated, such that stimuli rewarded in the past are favored in situations involving limited resources and competition. At the same time, task-irrelevant, high-arousal negative stimuli capture attention and divert resources away from tasks resulting in poor behavioral performance. Yet, investigations of how reward learning and negative stimuli affect perceptual and attentional processing have been conducted in a largely independent fashion. We have recently reported that performance-based monetary rewards reduce negative stimuli interference during perception. The goal of the present study was to investigate how stimuli associated with past monetary rewards compete with negative stimuli during a subsequent attentional task when, critically, no performance-based rewards were at stake. Across two experiments, we found that target stimuli that were associated with high reward reduced the interference effect of potent, negative distractors. Similar to our recent findings with performance-based rewards, our results demonstrate that reward-associated stimuli reduce the deleterious impact of negative stimuli on behavior.

## Introduction

At least two classes of paradigm have been used to investigate the effects of reward on perception and attention (Pessoa, in press; see also Della Libera et al., 2011; Camara et al., 2013). In a *proactive* paradigm, participants are informed that they will receive a reward during certain trials, while no reward is involved in others. Importantly, on each trial, trial type is indicated via an initial cue stimulus that precedes the target stimulus on which participants perform the task, thus allowing participants to engage goal-directed mechanisms. In addition, reward is often administered in a performance-contingent fashion. Proactive effects of reward include enhanced attentional filtering (Padmala and Pessoa, 2011), and improved working memory performance (Beck et al., 2010).

In a *reactive* paradigm, the possibility of reward is not cued in advance, and instead a specific stimulus feature is linked with reward. This means that participants cannot proactively engage in strategies that might enhance performance; instead, they can only react to stimulus features that are linked (or not) with reward. In reactive paradigms, in many cases, a training phase is used to associate certain items (or features) with reward. The training phase is followed by a subsequent task phase during which the effect of learning is evaluated. Moreover, the task is often performed under “extinction,” in other words, without any pairing between reward and the previously-paired feature. Studies employing reactive paradigms have demonstrated that learned stimulus-reward associations influence how attention is allocated, such that stimuli rewarded in the past are favored in situations involving limited resources and competition (Anderson, 2013; Chelazzi et al., 2013). For instance, stimuli paired with reward during an initial learning phase reduced the blink effect when presented as a second target stimulus during a subsequent attentional blink task (Raymond and O'Brien, 2009), and stimuli paired with reward in the past were easier to select as targets and harder to reject as distractors during a subsequent object identification task (Della Libera and Chelazzi, 2009).

A separate literature has investigated how aversive stimuli influence perceptual and attentional processing (Pessoa, 2002; Vuilleumier, 2005; Pourtois et al., 2013). These studies have established that task-irrelevant, high-arousal negative stimuli capture attention and divert resources away from the main task resulting in poorer behavioral performance (Pessoa, 2005). For instance, performance was impaired following negative stimuli during rapid visual stream tasks (Most et al., 2005), and discrimination of the orientation of peripheral bars was slower in the presence of central unpleasant images (Erthal et al., 2005). In all, negative stimuli capture attention much like reward-associated items discussed in the context of reactive paradigms.

In a recent study, we reported that, in a proactive manipulation of reward, the interference of negative items was eliminated (Padmala and Pessoa, 2014). Thus, when goal-driven mechanisms are engaged, reward is able to decrease the deleterious impact of aversive stimuli. The question addressed here was as follows: would a purely *reactive* manipulation of reward be able to counteract the interference by negative emotional stimuli, too? Two possible outcomes were anticipated. If negative items are too powerful, reward-associated items (via learning) would not be able to counteract them; in contrast, if the representation of reward-associated items is sufficiently strengthened, these items should be able to, at least in part, counteract negative items. Our goal was to adjudicate between these two alternatives.

**Figure 1:**
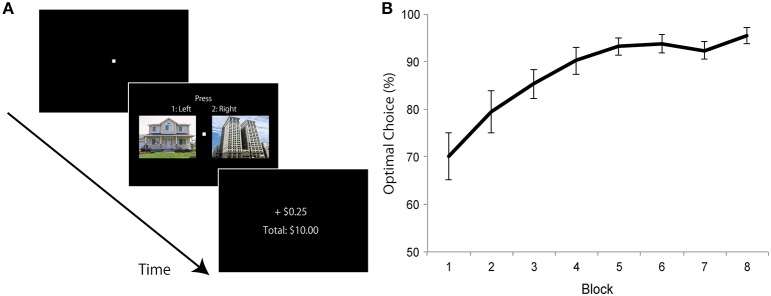
**Experiment 1: learning phase design and results. (A)** On each trial, one house and one building image were presented and participants were required to choose “left” or “right” image (not “house” or “building”). Immediately after a selection was made, they received feedback about the earnings. One category of images (e.g., “house”) was associated with a high probability (0.8) and the other category (e.g., “building”) was associated with a low probability (0.2) of winning reward. **(B)** Average probability of choosing the optimal image in each block. Error bars denotes standard error of the mean.

**Figure 2:**
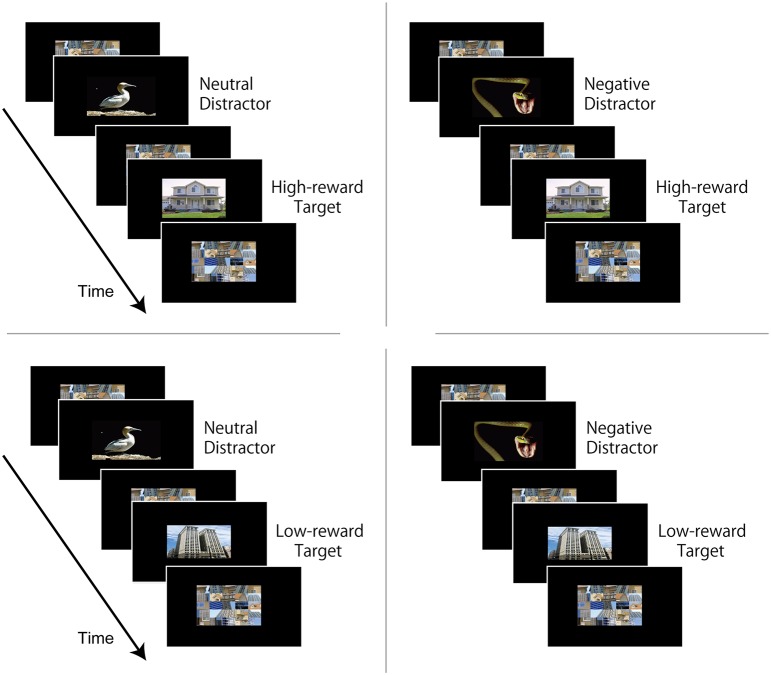
**Experiments 1 and 2: attentional task experiment design**. Each trial consisted of a rapid visual presentation of a stream of 17 images. The duration of each image was 83 ms for Experiment 1, and 83 ms or 100 ms in Experiment 2. The critical distractor image (neutral or negative) was positioned in the 4th, 6th, or 8th position in the visual stream. The target image that was paired with high-reward (e.g., “house”) or low-reward (e.g., “building”) in the learning phase always appeared two images after the critical distractor. The remaining 15 images were non-critical filler images presented in random order. Participants were instructed to ignore all images in the visual stream except the target image, and at the end of the visual stream they answered whether the target image was “house” or “building”.

To address our central question, in the present study, we sought to investigate how stimuli associated with monetary rewards during an initial learning phase compete with negative stimuli during a subsequent challenging visual task. It was previously shown that, during the rapid presentation of visual stimuli, the presence of a task-irrelevant high-arousal negative item interferes with performance (relative to a neutral stimulus), a pattern which is termed “emotion induced blindness” (Most et al., 2005). In the current study, we investigated the impact of reward learning on this kind of interference. To do so, during an initial learning phase, we paired a specific stimulus category (say “house”) with high-reward probability and another category (say “building”) with low-reward probability (Figure 1A). During a subsequent task, on each trial, the target stimulus was an image that, previously, was either paired with high or low reward probability during learning; target stimuli were preceded by a task-irrelevant image that was either neutral or negative (Figure 2). Importantly, the rapid serial visual presentation task was performed in the absence of monetary reward.

## Experiment 1

### Methods

#### Participants

Twenty-seven participants took part in Experiment 1 and provided written informed consent, as approved by the Institutional Review Board of the University of Maryland, College Park. Participants were free from psychiatric or neurological disease or related past history, as indicated via self-report. We excluded data from two participants from the analysis due to poor performance in the rapid visual stream task (at or below 50% chance performance in the “baseline” condition, namely, neutral distractor with low-reward target). Thus, the results reported here are based on data collected from 25 participants (15 female; 19.7 ± 1.5 (SD) years old).

#### Apparatus

Visual stimuli were displayed on a NEC Multisync FE992 CRT display with a resolution of 1024 × 768 pixels (refresh rate: 60 Hz). Visual display and data collection were controlled using the Psychophysics toolbox (Brainard, 1997) for the reward learning phase and Presentation software (http://www.neurobs.com/) for the rapid visual stream phase. A USB connected keypad was used to collect button responses. Participants were tested individually in a darkened room, and the viewing distance was approximately 50 cm.

#### Stimuli and task

##### Reward learning

A conventional reward learning paradigm was used (Raymond and O'Brien, 2009). On each trial, a white fixation cross (0.2° × 0.2°) was presented in the center of a uniform black background, and two color images (one house and one building: 10.4° × 8.3°) were presented to the left (10.3°) and right (10.3°) of the center of the screen (Figure 1A). Positions of house and building images varied across trials in a random manner. Participants were required to choose “left” or “right” image (not “house” or “building”) by pressing buttons 1 and 2 on the keypad. Images were displayed on the screen until a response was made. Immediately after a selection was made, they received feedback about the earnings for the trial and the cumulative total amount earned until that point in time (Figure 1A). Each trial ended with a 1500-ms blank screen. Monetary reward was 25 cents (default: zero cents) of “experimental money” on each trial. One category of images (say “house”) was associated with a high probability (0.8) of winning reward, and another category (say “building”) was associated with a low probability (0.2) of winning reward. Participants were neither informed about the category that was associated with high/low reward probability nor the actual reward probabilities. Participants were just asked to choose the left or right image to maximize their earnings on each trial. Assignment of high and low reward probability category type (“house” or “building”) was counterbalanced across participants. Eight blocks of 64 trials each (total of 512 trials) were employed. Thirty-two color images of houses and 32 of buildings were used, and each image was used twice in each block. At the end of the experiment, participants were paid 10% of the total experimental money they accrued as bonus cash; they were informed about this conversion factor prior to the start of the experiment. On average, participants won $9.02 of bonus cash (in addition to the base pay of $10).

##### RSVP task

Stimuli were color images and subtended 10.4° × 8.3°. Critical distractor images were 36 neutral and 36 negative pictures obtained mostly from the International Affective Picture System (IAPS) database (Lang et al., 2005), and supplemented by similar pictures that were used previously (Most et al., 2005; see Appendix). Target images were 36 house and 36 building images that included the same 32 used in the reward learning phase plus four new ones for each stimulus category. The non-critical distractor (“filler”) stimuli were 15 scrambled images that were generated by randomly mixing the visual content from house and building images.

Each trial consisted of a stream of 17 images at the center of the screen (Figure 2). The duration of each image was set to 83 ms. The critical distractor image was positioned in the 4th, 6th, or 8th position in the visual stream. Because previous studies observed robust interference effects at lag 2, the target image always appeared two images after the critical distractor (Most et al., 2005). The remaining 15 images were non-critical filler images presented in random order. Participants were instructed to ignore all images in the visual stream except the target image; at the end of the visual stream, they answered whether the target image was “house” or “building” as accurately as possible by pressing buttons 1 and 2 on the keypad. The trial ended with a 5000-ms blank screen. Three blocks of 48 trials were employed (total of 144 trials). Each block contained 12 trials per condition (equally distributed across three distractor positions) presented in random order, yielding a total of 36 trials per condition across three blocks. No feedback about performance was provided. Participants were explicitly informed that no monetary reward was at stake during the rapid visual stream task. Across the experiment, neutral and negative images were repeated once; they were shown once on trials in which the target category was associated with high-reward probability during learning (e.g., “house”), and once on trials in which with the target category was associated with low-reward probability during learning (e.g., “building”). Hence, there were four trial types (Neutral distractor with high-reward target, Negative distractor with high-reward target, Neutral distractor with low-reward target, and Negative distractor with low-reward target; see Figure 2), allowing us to investigate the interactions between *reward learning* and *distractor* interference.

The three main experimental blocks were preceded by a short practice block with 12 rapid visual stream trials. Performance feedback (“correct”/“incorrect”) was provided after each trial in the practice block.

### Data analysis

#### Reward learning task

As done previously (Raymond and O'Brien, 2009), we assessed learning by calculating the proportion of time participants chose the high-reward probability stimulus in each block. To further quantify learning, we compared the proportion of high-reward category selection between the first and last blocks using a paired *t*-test (the arcsine transformation was employed on proportion data to better meet the requirements of the *t*-test; that is, normally distributed data with equal variance).

#### Rapid visual stream task

Because RT data are less interpretable given that participants made their response only at the completion of the visual stream, we focused on accuracy data as in previous studies (Most et al., 2005; Smith et al., 2006). For each participant, mean accuracy rate data were determined as a function of stimulus value (high-reward, low-reward) and critical distractor type (neutral, negative) and a repeated-measures ANOVA was conducted. We used an alpha level of 0.05 for all statistical tests.

### Results and discussion

#### Reward learning task

Choice behavior during learning revealed a gradual increase in the proportion of high-reward category selection (Figure 1B). A paired *t*-test between choices during the first and last blocks revealed increased high-reward category selection during the last block compared to the first one [*t*_(24)_ = 5.728, *p* < 0.0001, *d* = 1.42].

#### Rapid visual stream task

**Figure 3:**
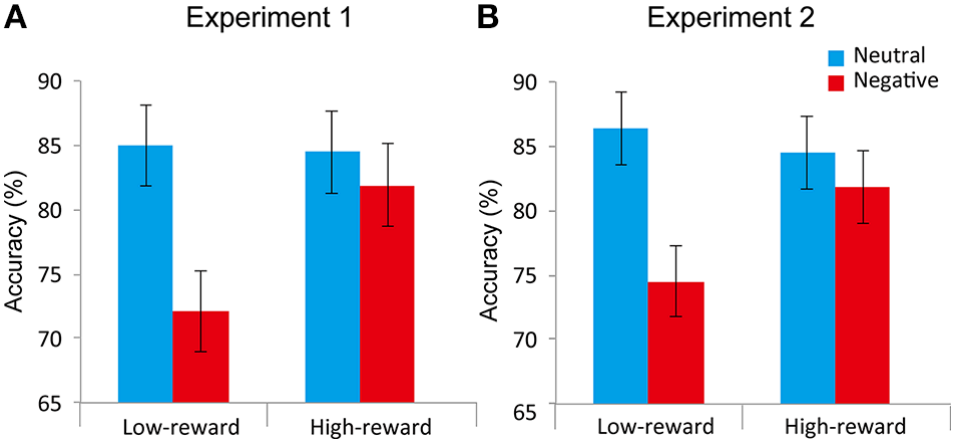
**Attentional task results of Experiment 1 (A) and Experiment 2 (B)**. During low-reward target trials, negative distractors decreased accuracy relative to neutral ones. This difference was reduced during the high-reward target condition. Error bars denote the standard within-subject error term for interaction effects (Loftus and Masson, 1994).

Accuracy data (Figure 3A) were evaluated according to a 2 *Value* (low-reward, high-reward) x 2 *Distractor* (neutral, negative) repeated-measures ANOVA. The main effect of *Distractor* was robust [*F*_(1, 24)_ = 30.399, *p* < 0.0001, η^2^_*p*_ = 0.559]. The mean accuracy rate during the negative condition (76.99%) was lower compared to the neutral (84.72%) condition replicating the previously reported emotional interference effect (McHugo et al., 2013). The main effect of *Value* was not detected [*F*_(1, 24)_ = 1.576, *p* = 0.22, η^2^_*p*_ = 0.062]. Critically, an interaction between *Value* and *Distractor* was observed [*F*_(1, 24)_ = 7.738, *p* = 0.010, η^2^_*p*_ = 0.244]. To understand the nature of the two-way interaction, we ran two additional paired *t*-tests (neutral vs. negative) separately for low- and high-reward conditions. We detected a negative distractor effect during the low-reward condition (12.89%; *t*_24_ = 5.189, *p* < 0.0001, *d* = 0.71), but not during the high-reward condition (2.55%; *t*_24_ = 1.185, *p* = 0.248, *d* = 0.14). In addition, we ran separate paired *t*-tests (low- vs. high-reward) for the neutral and negative conditions. We observed an effect of reward in the negative condition (9.78%; *t*_24_ = 2.117, *p* < 0.005, *d* = 0.50), but not in the neutral condition (−0.56%; *t*_24_ = 0.157, *p* = 0.877, *d* = 0.03).

The results of Experiment 1 show that stimuli associated with high reward reduced the interference effect of negative stimuli on task performance. However, during learning, participants selected high-reward stimuli more often than low-reward stimuli, which might have led to enhanced attention to high-reward stimuli during the subsequent task phase. In other words, it is conceivable that the observed reward effect during the visual stream task was due to the frequency of selection, and not from value learning itself. Experiment 2 aimed to rule out this possibility, as described next.

## Experiment 2

**Figure 4:**
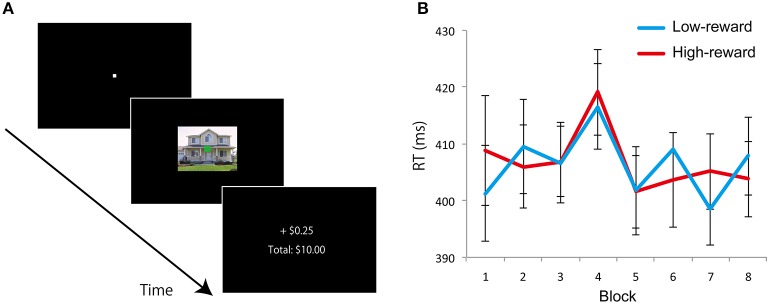
**Experiment 2: Learning phase design and results. (A)** On each trial, an image of house or building with an overlaid color fixation square (yellow or green) was presented and participants were required to judge the color of the fixation square. Immediately after a response was made, they received feedback about the earnings. Accurate and fast trials with one category of background images (e.g., “house”) was associated with a high probability (0.8) and the other category (e.g., “building”) was associated with a low probability (0.2) of winning reward. **(B)** Mean RT of correct trials with low-reward and high-reward background image in each block. Error bars denotes standard error of the mean.

In Experiment 1, reward learning was task relevant as participants made a choice between two stimulus categories that were associated with high- and low-reward probabilities. In Experiment 2, to attempt to equalize the frequency of selection, the stimulus categories associated with reward were dissociated from the behavioral task. On each trial, a house or building image was presented with an overlaid yellow or green fixation square, and participants were asked to discriminate the color of the fixation square (Figure 4A). As in Experiment 1, the probability of winning monetary reward was associated with the house or building categories, but unlike in Experiment 1, the behavioral task was unrelated to the stimulus categories. Thus, if the behavioral effect observed in Experiment 1 was due to the selection history during the learning phase, to the extent that choice frequency was matched in Experiment 2, the behavioral effect should be eliminated. Finally, note that the reward manipulation in Experiment 2 was task irrelevant; we made no attempt to carefully characterize awareness, as our goal was solely to attempt to match choice frequency.

### Methods

#### Participants

Twenty-seven new participants took part in Experiment 2 and provided written informed consent, as approved by the Institutional Review Board of the University of Maryland, College Park. Participants were free from psychiatric or neurological disease or related past history, as indicated via self-report. Data from three participants were excluded from the analysis because of poor performance in the rapid visual stream task (at or below 50% chance performance in the baseline condition, namely, neutral distractor with low-reward target). Thus the results reported in this study are based on data collected from 24 participants [16 female; 19.9 ± 1.3 (SD) years old].

#### Apparatus

The apparatus in Experiment 2 was the same as described in Experiment 1.

#### Stimuli and task

##### Reward learning

On each trial, an image (house or building: 10.4° × 8.3°) with an overlaid colored fixation square (yellow or green: 0.4° × 0.4°) was presented at the center of the display (Figure 4A). The pairing of fixation square color with house/building image was randomized across trials. Participants were instructed to judge whether the color of the fixation square was yellow or green as accurately and quickly as possible by pressing buttons 1 or 2 on the keypad. Images were displayed on the screen until a response was made. Immediately after the response was made, they received feedback about the earnings for the trial and the cumulative total amount earned until that point in time (Figure 4A). Each trial ended with a 1500-ms blank screen. When participants responded accurately and fast (RT < 600 ms), they had a chance to win 25 cents (default: zero cents) of “experimental money” on each trial based on the category of the background image (not the fixation square color). One image category (e.g., “house”) was associated with a high probability (0.8) of winning 25 cents, and another category (e.g., “building”) was associated with a low probability (0.2) of winning 25 cents. Participants were neither informed about the background category that was associated with high or low reward probability nor the actual reward probabilities. Participants were just asked to respond fast and accurately to the fixation square color to maximize their earnings on each trial. Assignment of high- and low-reward probability category (“house” or “building”) was counterbalanced across participants. Eight blocks of 72 trials (total of 576 trials) were employed. Thirty-six color images of houses and 36 of buildings were used, and each image was used once in each block. At the end of the experiment, participants were paid 10% of the total experimental money they accrued as bonus cash; they were informed about this conversion factor prior to the start of the experiment. On average, participants won $6.75 of bonus cash (in addition to the base pay of $10) in this experiment.

##### Rapid visual stream task

The task was the same as in Experiment 1, except that the duration of each image in the visual stream was set to 83 or 100 ms depending on performance in the practice block. If accuracy in the practice block was equal to or more than 75% (i.e., at least 9 corrects out of 12), the duration of each image in the main experimental blocks was kept at 83 ms; otherwise it was set to 100 ms. Based on this criterion, the duration of each image in the main experimental blocks was set to 100 ms for three participants and 83 ms for all other participants. House and building images were the same as those used in the reward learning phase.

### Data analysis

#### Reward learning task

Unlike in Experiment 1, the behavioral task was unrelated to the background stimulus categories (participants judged the color of the overlaid fixation square). Thus, as done previously (Wang et al., 2013), we assessed learning by comparing RTs on correct trials with high- and low-reward probability backgrounds. RTs more than 3 SD away from the mean value for each participant and each condition (1.28% trials on average) were excluded from the analysis. To further quantify the learning, we compared the RT value between high and low-reward category trials during the last block using a paired *t*-test.

#### Rapid visual stream task

The visual stream task in Experiment 2 was the same as Experiment 1.

### Results and discussion

#### Reward learning task

The RT data during the learning phase did not reveal a discernible learning pattern (Figure 4B), as the paired *t*-test between high- and low-reward category trials during the last block did not reveal a significant difference [*t*_(23)_ = 1.294, *p* = 0.208, *d* = 0.12]. We further probed the results based on behavioral choices. In terms of accuracy, namely the color of the fixation square (green and yellow), across all eight blocks, both conditions had nearly identical values around 94% correct given that the task was very easy (the one exception was during block four, where performance was better for green than yellow; but given eight comparisons, it is not surprising that one would have a “significant” difference). We also analyzed choices based on the category of the task-irrelevant stimulus category. In other words, when participants indicated “green,” what percentage was when the background was a house and what percentage was when the background was a building? Likewise, when participants indicated “yellow.” When sorted this way, choices were nearly exactly 50% for each stimulus category for each of the eight blocks. Together, based on RT and behavioral choice, no evidence for a reward learning effect was evident in Experiment 2.

#### Rapid visual stream task

Accuracy data (Figure 3B) were evaluated according to a 2 *Value* (low-reward, high-reward) x 2 *Distractor* (neutral, negative) repeated-measures ANOVA. The main effect of *Distractor* was robust [*F*_(1, 23)_ = 17.870, *p* < 0.0005, η^2^_*p*_ = 0.437]. The mean accuracy rate during the negative condition (78.18%) was lower compared to the neutral (85.42%) condition replicating the previously reported emotional interference effect (McHugo et al., 2013). The main effect of *Value* was not detected [*F*_(1, 23)_ = 1.367, *p* = 0.25, η^2^_*p*_ = 0.056]. Critically, an interaction between *Value* and *Distractor* was observed [*F*_(1, 23)_ = 7.505, *p* = 0.012, η^2^_*p*_ = 0.246]. To understand the nature of the two-way interaction, we ran two additional paired *t*-tests (neutral vs. negative) separately for the low- and high-reward conditions. We detected a negative distractor effect during the low-reward condition (11.80%; *t*_23_ = 5.274, *p* < 0.0005, *d* = 0.70), but not during the high-reward condition (2.66%; *t*_23_ = 1.051, *p* = 0.30, *d* = 0.17). Moreover, we conducted separate paired *t*-tests (low- vs. high-reward) for the neutral and negative conditions. We detected an effect of reward in the negative condition (7.29%; *t*_24_ = 2.450, *p* < 0.005, *d* = 0.43), but not in the neutral condition (−1.85%; *t*_24_ = 0.674, *p* = 0.507, *d* = 0.12).

We conducted Experiment 2 to assess the possibility that the observed reward effects in Experiment 1 were due to differences in selection frequency between low- and high-reward associated stimuli during learning phase. Since selection history was equated during learning, choice frequency cannot explain the results of Experiment 1. Furthermore, selection history (for a related, though different concept of “history,” see Awh et al., 2012) is less likely to have been the main determinant of the behavioral pattern in Experiment 1, which was similar to that of Experiment 2.

## General discussion

In the present study, we investigated how visual items that acquired motivational significance during reward learning compete with negative stimuli during a subsequent attentional task. Across two experiments, stimuli associated with past high reward reduced the effect of task-irrelevant negative stimuli.

Negative stimuli, such as high-arousal unpleasant images, exhibit interference effects when presented as task-irrelevant distractors during perceptual and attentional tasks (Hartikainen et al., 2000; Erthal et al., 2005; MacNamara and Hajcak, 2009). We evaluated whether target stimuli associated with reward in the past would reduce this interference during a subsequent visual task. We used two different versions of reward learning across two experiments. In Experiment 1, reward learning was relevant to the task, whereas in Experiment 2 it was not. In both experiments we observed a similar pattern of results, where stimuli associated with high reward reduced the interference effect of negative stimuli on task performance.

In the standard attentional blink (Raymond et al., 1992), participants are required to perform two tasks on each trial, one for each target stimulus. The blink effect typically observed is critically dependent on the first task and has not been observed when participants passively view the first target (Raymond et al., 1992). In the rapid visual stream task we used in this study (Most et al., 2005), participants are asked to detect a single target; there is no task related to the preceding neutral/negative images. At the present time, the precise mechanisms underlying interference by negative items during the task employed here are unclear. Some researchers have suggested that the mechanisms are actually distinct from those involved in the standard attentional blink paradigm (Wang et al., 2012; McHugo et al., 2013), whereas others propose that similar processes are involved (Kennedy et al., 2014).

**Figure 5:**
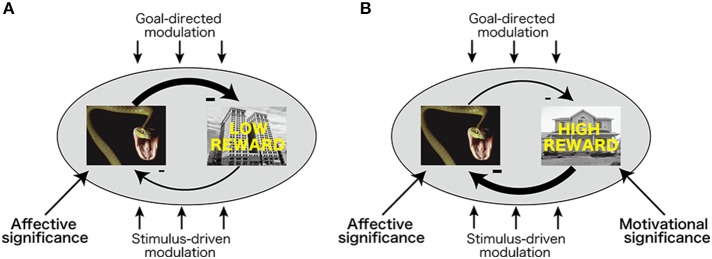
**Competition between affectively and motivationally significant stimuli. (A)** During competition between high-arousal task-irrelevant negative stimuli and task-relevant stimuli with low motivational significance, negative stimuli dominate, thereby leading to interference. **(B)** During competition between task-irrelevant high-arousing negative stimuli and task-relevant stimuli with high motivational significance, motivationally significant stimuli more effectively compete with negative stimuli.

The processing of affectively significant stimuli such as high-arousal negative pictures is enhanced in visual cortex (Lang et al., 1998; Schupp et al., 2003), allowing them to more effectively compete and win the competition against neutral stimuli (Pessoa, 2002). In a related fashion, Most and colleagues (Wang et al., 2012) have proposed that the competition between task-irrelevant emotional stimuli and target (neutral) stimuli during perceptual processing is responsible for the interference effects observed here. They suggest that emotional (relative to neutral) task-irrelevant stimuli dominate over the perceptual representation of spatiotemporally adjacent task-relevant (neutral) stimuli, making the latter win the competition less frequently (Figure 5A; Most and Wang, 2011). In the current study, pairing with reward in the past enhanced the motivational significance of high-reward target stimuli (Hickey et al., 2010; Anderson et al., 2011), possibly allowing them to more effectively compete with negative stimuli in visual cortex resulting in being detected (Figure 5B).

The present findings do not rule out alternative interpretations, in particular those related to more “central” processing stages. Similar to the mechanisms suggested in the two-stage model of the standard attentional blink paradigm (Chun and Potter, 1995), it has been suggested that in the rapid visual stream task used here, task-irrelevant emotional stimuli could gain access to working memory and interfere with the consolidation of temporally adjacent task-relevant (neutral) stimuli into working memory (Kennedy et al., 2014). It is thus relevant that a recent study reported that reward-associated visual features have enhanced representation in working memory (Gong and Li, 2014). Thus, an alternative interpretation of our results is that high-reward target stimuli offset the interference from negative stimuli at more central stages of working memory processes.

The results of the current study are related to additional findings from our lab (Hu et al., 2013; Padmala and Pessoa, 2014) that have shown reduced negative stimuli interference in the presence of *performance-based* monetary rewards. In one study (Padmala and Pessoa, 2014), participants performed an orientation discrimination task on peripheral bars while ignoring centrally presented task- irrelevant neutral or negative pictures. Motivation was manipulated on each trial by presenting a reward or no-reward cue before the task phase, which informed participants about the chance of earning additional money based on fast and accurate performance. In another study (Hu et al., 2013), participants performed a discrimination task on two stimulus types that were overlaid on a background color that was previously paired (CS+) or unpaired (CS−) with shock. Motivation was manipulated by associating one of the foreground stimulus types with *performance-based* reward and the other with no-reward. In both studies, behaviorally we observed reduced task-irrelevant negative picture interference under reward conditions. It has been argued that a drawback of performance-based reward studies is that the observed behavioral effects cannot be unequivocally ascribed to reward-based motivation or increased attention (Maunsell, 2004; but see Pessoa (2013) for a discussion of problems linked to trying to disentangle motivation and attention). In the current study, negative interference was reduced while no performance-based monetary rewards were at stake during the main task. This reduction of negative interference based on the association of target stimuli with past reward illustrates the interaction between reward processing and negative emotion. The results of Experiment 2 are particularly noteworthy as the reward manipulation, while task-irrelevant, still reduced interference effects during the subsequent unrewarded visual task.

In Experiment 2, we found that a task-irrelevant manipulation of reward led to a behavioral effect that was similar to the one observed with the task-relevant manipulation in Experiment 1. This raises the question of whether the reward manipulation in Experiment 2 was “implicit.” We stress that it was not our objective to employ an “unaware” manipulation of reward, and we made no attempt to characterize it in terms of awareness. We informally asked participants if they had noticed a discernible pattern of reward association, but the assessment was not systematic enough to qualify as an evaluation of *subjective* awareness (Kunimoto et al., 2001; Szczepanowski and Pessoa, 2007). And, of course, we did not attempt to evaluate *objective* awareness with a separate forced-choice task, for instance (Kunimoto et al., 2001; Szczepanowski and Pessoa, 2007). In all, our interpretation of the findings of Experiment 2 is agnostic regarding awareness. In fact, it is often the case that when awareness is eliminated based on objective criteria, unaware effects disappear (Holender, 1986; Pessoa, 2005).

In conclusion, we investigated the effects of reward learning on negative picture interference during an attentional task. We found that stimuli associated with reward in the past reduced the interference effect of potent negative stimuli. Here, we reported that the deleterious impact of negative stimuli on behavior was reduced in situations that involved competition with stimuli that previously acquired positive motivational significance.

### Conflict of interest statement

The authors declare that the research was conducted in the absence of any commercial or financial relationships that could be construed as a potential conflict of interest.

## Appendix

**List of images taken from the IAPS database Lang et al., 2005**:

**21 negative images** (mean valence = 1.76, *SD* = 0.45; mean arousal = 6.61, *SD* = 0.60)

1050, 3000, 3010, 3015, 3030, 3053, 3060, 3061, 3062, 3063, 3064, 3071, 3100, 3102, 3110, 3120, 3130, 3168, 3170, 3301, 6350

**33 neutral images** (mean valence = 5.71, *SD* = 0.90; mean arousal = 3.60, *SD* = 0.65)

1450, 1500, 1600, 1670, 2037, 2358, 2370, 2372, 2373, 2383, 2393, 2396, 2410, 2480, 2485, 2487, 2500, 2515, 2560, 2570, 2575, 2580, 2590, 2600, 2620, 2702, 2749, 2840, 2850, 2870, 4100, 4533, 4536

**Other images from Most et al., 2005 study:**

**15 negative images^*^** (mean valence = 1.71, *SD* = 0.53; mean arousal = 6.27, *SD* = 0.52)

**3 neutral images^*^** (mean valence = 4.98, *SD* = 0.10; mean arousal = 2.81, *SD* = 0.35)

^*^ Ratings were provided by Dr. Steven Most.
